# The Importance of Heat Shock-Related 70-kDa Protein 2 Expression in Laryngeal Squamous Cell Carcinomas

**DOI:** 10.5152/eurasianjmed.2022.21113

**Published:** 2022-06-01

**Authors:** Onur Ceylan, Remzi Arslan

**Affiliations:** 1 Department of Pathology, Atatürk University, Faculty of Medicine, Erzurum, Turkey

**Keywords:** Laryngeal squamous cell carcinoma, heat shock proteins, HSPA-2, immunoreactivity, prognostic importance

## Abstract

**Objective:** Laryngeal squamous cell carcinoma has a high mortality rate, and approximately 60% of patients are in advanced stage at the time of diagnosis. Thus, determining the prognostic parameters of this cancer and markers related to these parameters are very important. There are studies showing that heat shock-related 70-kDa protein 2, which is used as a biomarker, may be associated with prognostic parameter in some cancers. However, no study has investigated the prognostic role of heat shock-related 70-kDa protein 2 in laryngeal squamous cell carcinoma in the literature. Thus, in our study, the aim was to examine the relationship between heat shock-related 70-kDa protein 2 expression and clinicopathological prognostic parameters in laryngeal squamous cell carcinoma.

**Materials and Methods:** Our study included 104 patients diagnosed with laryngeal squamous cell carcinoma between January 2005 and January 2016. The correlation between heat shock-related 70-kDa protein 2 expression and prognostic parameters was investigated by using the immunohistochemical method with heat shock-related 70-kDa protein 2 antibody.

**Results:** In all the cases, heat shock-related 70-kDa protein 2 positivity was determined in tumoral areas (100%). The overexpression rate of heat shock-related 70-kDa protein 2 in adjacent non-cancerous tissues with dysplasia was 48/104 (42%) (*P* < .0001). A significant relationship was found between heat shock-related 70-kDa protein 2 expression and important prognostic parameters such as macroscopic tumor size, lymphovascular invasion, primary tumor, lymph node metastasis, distant organ metastasis, tumor, node, metastasis (TNM) stage stage, recurrence, and survival rates (*P* < .05).

**Conclusion:** Our study supports the presence of an association between high heat shock-related 70-kDa protein 2 expression levels and prognostic parameters in laryngeal squamous cell carcinoma. We consider that heat shock-related 70-kDa protein 2 can be used as a prognostic marker in laryngeal squamous cell carcinoma. In addition, it may be important in early diagnosis due to its increased expression even under laryngeal squamous cell carcinoma precursor conditions.

**Main Points**

- The expression of heat shock-related 70-kDa protein 2 (HSPA-2) is involved in carcinogenesis, tumor development, progression, and metastasis in laryngeal squamous cell carcinoma (LSCC).
- The expression of HSPA-2 is an important prognostic parameter in LSCC.
- The expression of HSPA-2 may be important in the early diagnosis of LSCC.

## Introduction

Laryngeal cancer constitutes 30%-40% of head and neck malignancies and 1%-2.5% of all malignant cancers.^1^ Every year, head and neck cancers are seen in more than half a million people worldwide, and one-fourth of these cases consist of laryngeal squamous cell carcinomas (LSCCs).^2^ According to the GLOBOCAN 2018 data, the incidence and mortality rates of laryngeal cancer (100 000 per person, standardized rates according to age) are 3.6/100 000 and 1.9/100 000, respectively, in males and 0.5/100 000 and 0.3/100 000, respectively in females.^3^ It has been reported that laryngeal cancer is seen 7 times more frequently in males, and it makes up 1% of deaths related to malignancies in the population and 1.6% in males.^4^ Laryngeal carcinomas are 95%-98% squamous cell carcinomas (SCC), and it takes the second place after lung cancer among respiratory system malignancies.^2^

Laryngeal squamous cell carcinoma has a high mortality rate, and approximately 60% of patients are in advanced stage (stages III and IV) at the time of diagnosis.^5^ Although the incidence of laryngeal cancer has declined, the 5-year survival rate has only slightly dropped, from 66% to 63%, within the last 40 years.^6^ When all these factors are considered, it is very important to understand the pathogenesis of this cancer and determine its prognostic parameters and markers related to these parameters.

There are studies showing that heat shock proteins (HSP), which are used as biomarkers, may be associated with prognostic parameters in some cancers.^7-9^ Heat shock proteins refer to a large chaperon protein group comprising a few different subgroups according to certain characteristics.^10^ They have protective function for cells by creating HSP adaptation mechanisms and constitute an important step in the defense system.^11^ Heat shock-related 70-kDa protein (HSPA/HSP70), is the largest group of HSPs, with the genes for this family displaying many different functions, localization, and expression.^10^ HSPA-2, also known as HSP70-2, is a significant member of the HSPA family but has not yet been well studied. It was first defined as a protein specific to the testicles having a fundamental role in primary spermatocytes and spermatogenesis in spermatids.^12^ In addition, it has been reported that HSPA-2 can be seen in non-testicular tissues and plays an important role in carcinogenesis as well as tumor growth.^13^

In the literature, there are various studies on this topic in different organs.^7-9,14-17^ However, the number of studies on the prognostic role of HSPA in LSCC is very low.^11^ In addition, we did not find any study investigating the prognostic role of HSPA-2 in LSCC in the literature. Therefore, in our study, we aimed to examine the relationship between HSPA-2 expression and clinicopathological prognostic parameters in LSCC.

## Materials and Methods

### Patients’ General Information and Tissue Specimens

**Figure 1.**
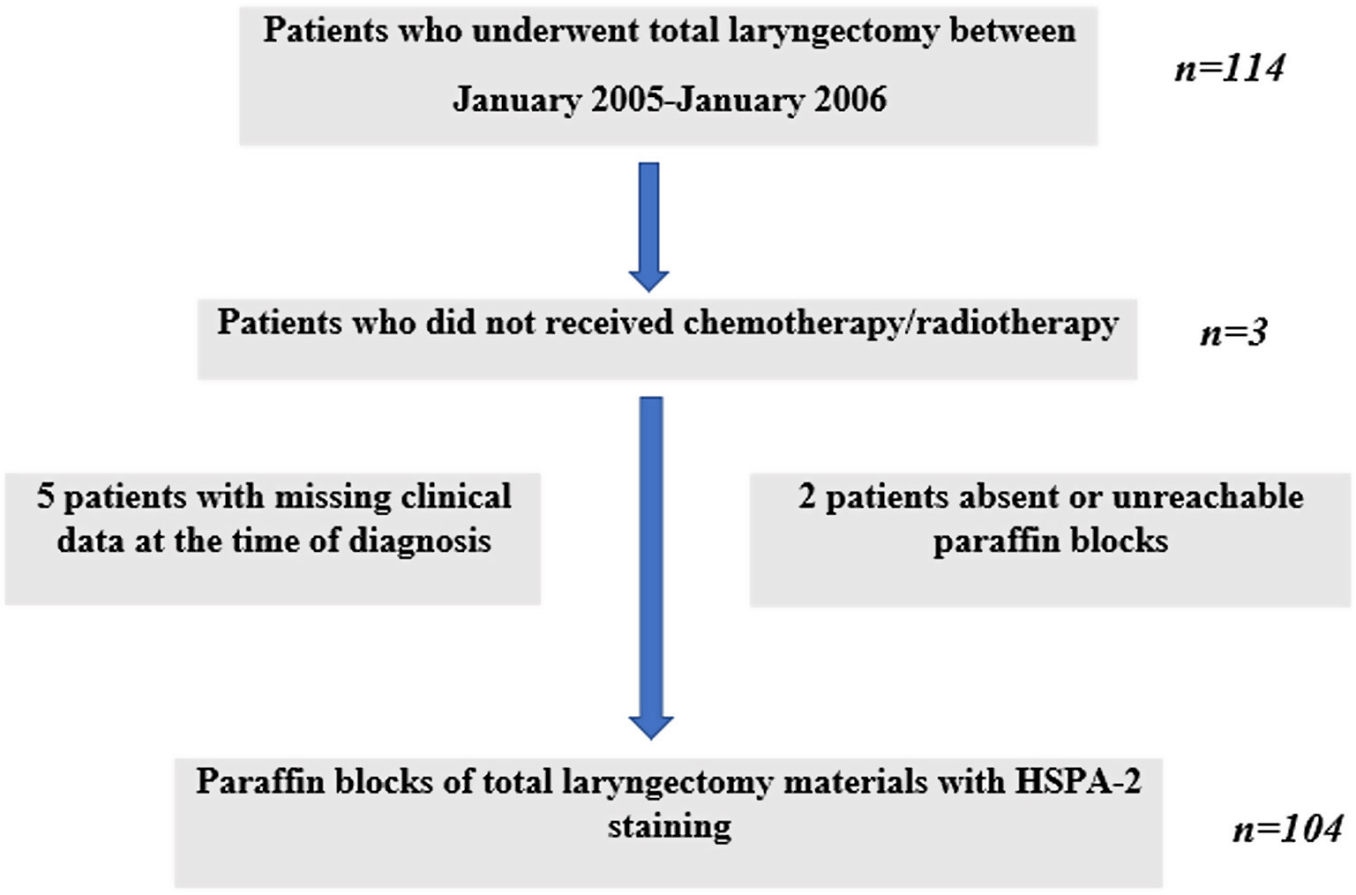
Study flowchart showing the included and excluded patients.

After receiving approval from the local ethics committee for the study (08-31/December 26, 2019), all laryngectomy materials examined between January 2005 and January 2016 were investigated retrospectively. Before the examination, consent was obtained from the cases for the use of the material. Patients who had received chemotherapy/radiotherapy, those with missing clinical data at the time of diagnosis, and those for whom paraffin slides could not be reached were excluded from the study. A total of 104 patients were selected for the final evaluation (Figure 1). The clinical information of the patients was obtained from the automation system of our hospital. Prognostic parameters including macroscopic tumor size, lymphovascular invasion, primary tumor (pT), lymph node metastasis (pN), distant organ metastasis (pM), tumor, node, metastasis (TNM) stage, recurrence, and survival rates were obtained for each patient and recorded. The staging was made according to the TNM staging system (American Joint Committee on Cancer).^18^ Recurrence and metastasis were determined by imaging methods, clinical examination, epicrisis, and examination of pathological materials sent to our department in the postoperative period. In order to determine the survival, recurrence, and metastasis rates, the patients were followed up for 160 months, with a median duration of 32.3 months. Changes after 60 months were not taken into account. Survival was evaluated from the start of treatment to the date of death or survival status on the last date of follow-up. The pathology reports, slides, and paraffin blocks of the patients were obtained from our archive. Paraffin blocks containing adjacent non-cancerous tissues of at least 1 cm in length were selected for the immunohistochemical study.

### Immunohistochemistry

Sections of 4 µm were taken from the blocks where the tumor was the densest and placed in the Roche Ventana automatic immunohistochemistry staining device (Ventana Medical Systems, Tucson, AZ, USA) after drying in 70˚ for 15 minutes. The tissues in the device were treated with ULTRA Cell Conditioning Solution, hydrogen peroxide, and HSPA-2 antibodies (Nova Castra, Leica, Newcastle, United Kingdom) after being subjected to deparaffinization and dehydration processes.

The immunohistochemical stains were evaluated by 2 pathologists. The adjacent squamous epithelium not containing dysplasia was accepted as the negative control. Nuclear and/or cytoplasmic staining was considered positive for HSPA-2. The staining rate of HSPA-2 was evaluated as grade 0 (0; no staining), grade 1 (1; 1%-10% staining), grade 2 (2; 11%-49% staining), and grade 3 (3; ≥50% staining). In addition, the staining intensity of HSPA-2 was classified as grade 0 (0; no staining), grade 1 (1+; weak cytoplasmic and nuclear staining), grade 2 (2+; moderate cytoplasmic and nuclear staining), and grade 3 (3+; strong cytoplasmic and nuclear staining). The immunoreactivity score (histoscore) was calculated using the method of multiplying the intensity and rate. The histoscore values for HSPA-2 were evaluated as follows: negative (grade 0); 1-3, weak (grade 1); 4-6, moderate (grade 2); and 7-9, strong (grade 3).^15^

### Statistical Analysis

Whether the data fit normal distribution was investigated with the D’Agostino Pearson test. The data that were not normally distributed were presented as median values. The levels of HSPA2 in infiltrative areas and adjacent non-cancerous tissue were examined with the chi square test. The correlations of HSPA-2 expression with prognostic parameters were assessed using the Spearman correlation test. The Kaplan–Meier survival analysis was performed, and the log rank test was undertaken for the comparison of HSPA-2 expression between the groups. The Cox regression multivariate analysis was performed to identify independent prognostic factors. For the 2-tailed test, *P* value <.05 was accepted as significant. Statistical analyses were carried out using the MedCalc software (Ver 16, Ostend, Belgium).

## Results

### Patients’ Demographic and Histopathological Features

The mean age of the 104 total cases included in our study was determined as 56 ± 8.1 (33-82) years; 94 of the cases were male (90.3%), and the male/female ratio was 9.4/1. Glottic cancers (75.4%) were more common than supraglottic cancers (22%), and 3 primary subglottic cancers were recorded (2.6%). The average macroscopic radius of the tumor was determined as 3.1 ± 1.8 (0.8-6 cm) cm. Fifty-one (49%) of the patients died within 5 years of follow-up. All the patients were treated with surgical and integrated (chemotherapy and radiotherapy) treatments. The survival rate ranged from 1 to 52 months. The demographic and histopathological features of the cases are presented in Table 1.

### Expression of Heat Shock-Related 70-kDa Protein 2 in Different Areas of Laryngeal Tissue

**Figure 2.**
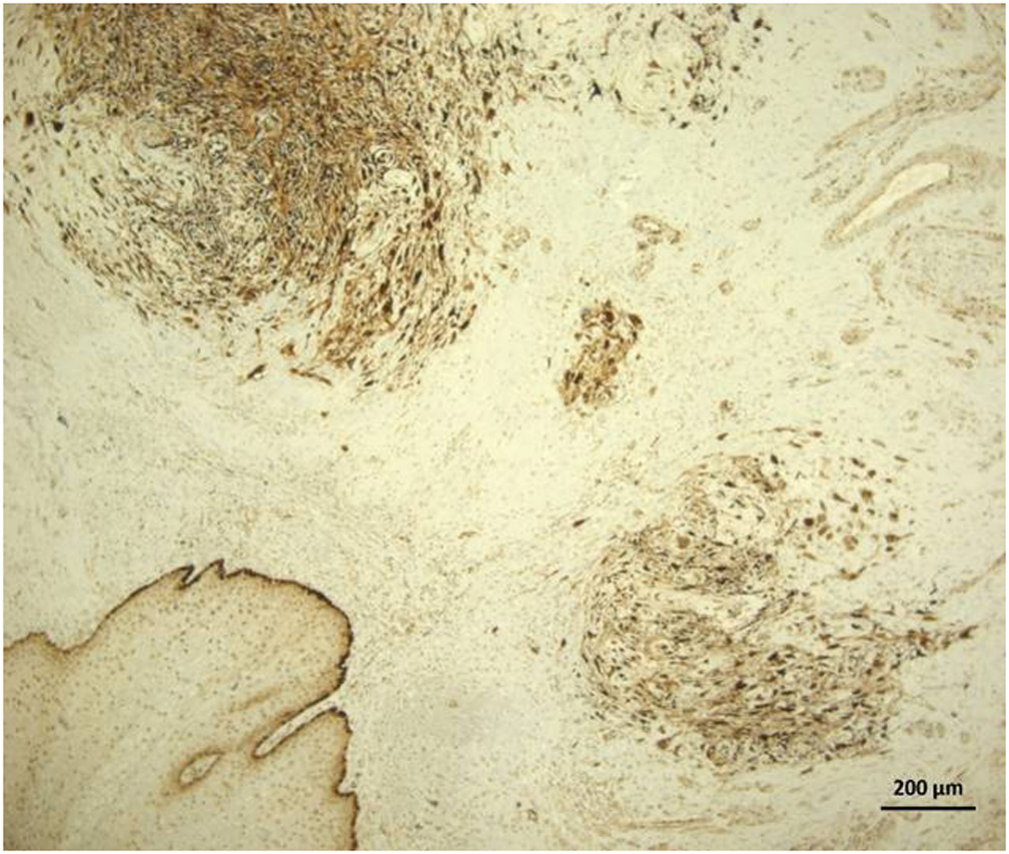
Heat shock-related 70-kDa protein 2 expression only in the basal layer of the adjacent epithelium and intense expression in areas with loss of differentiation.

**Figure 3.**
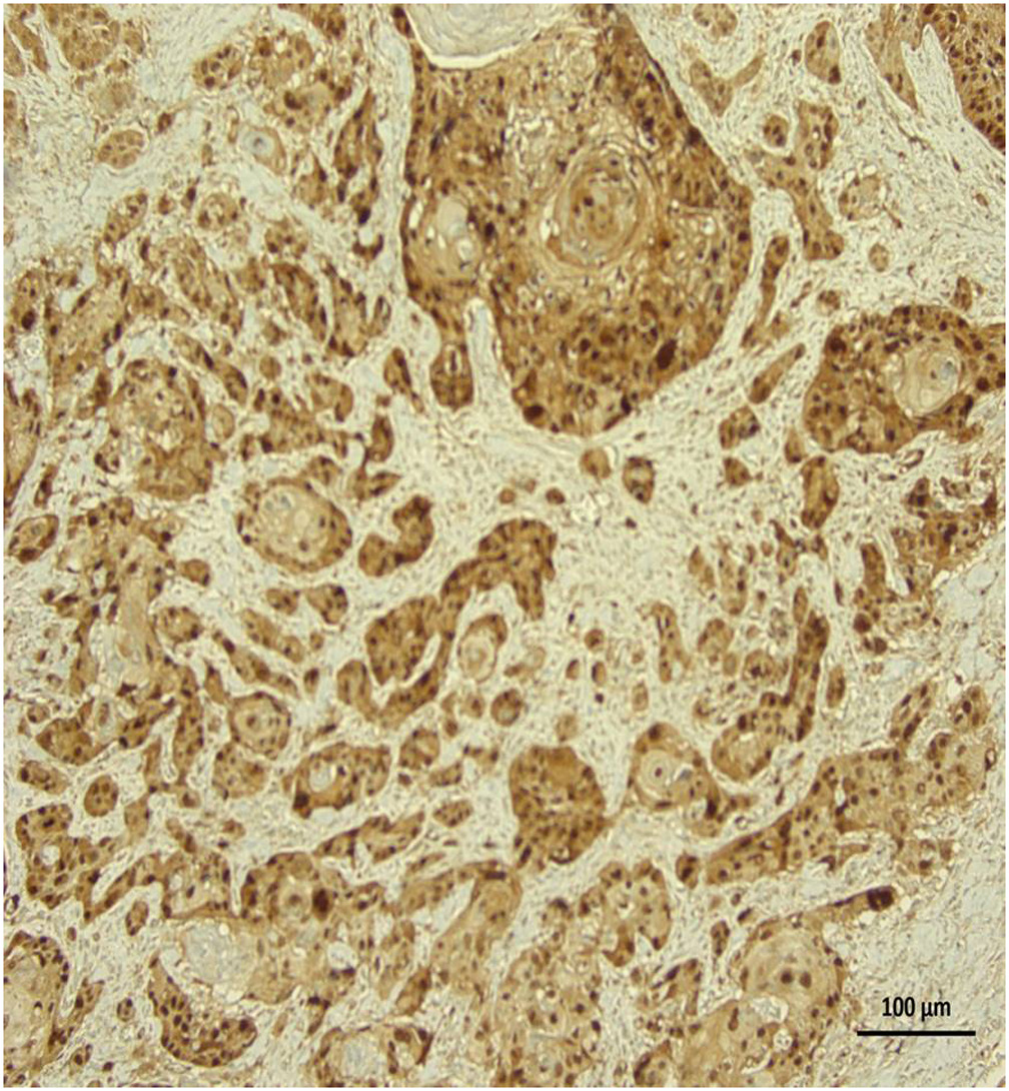
Heat shock-related 70-kDa protein 2 expression in infiltrative areas.

**Figure 4.**
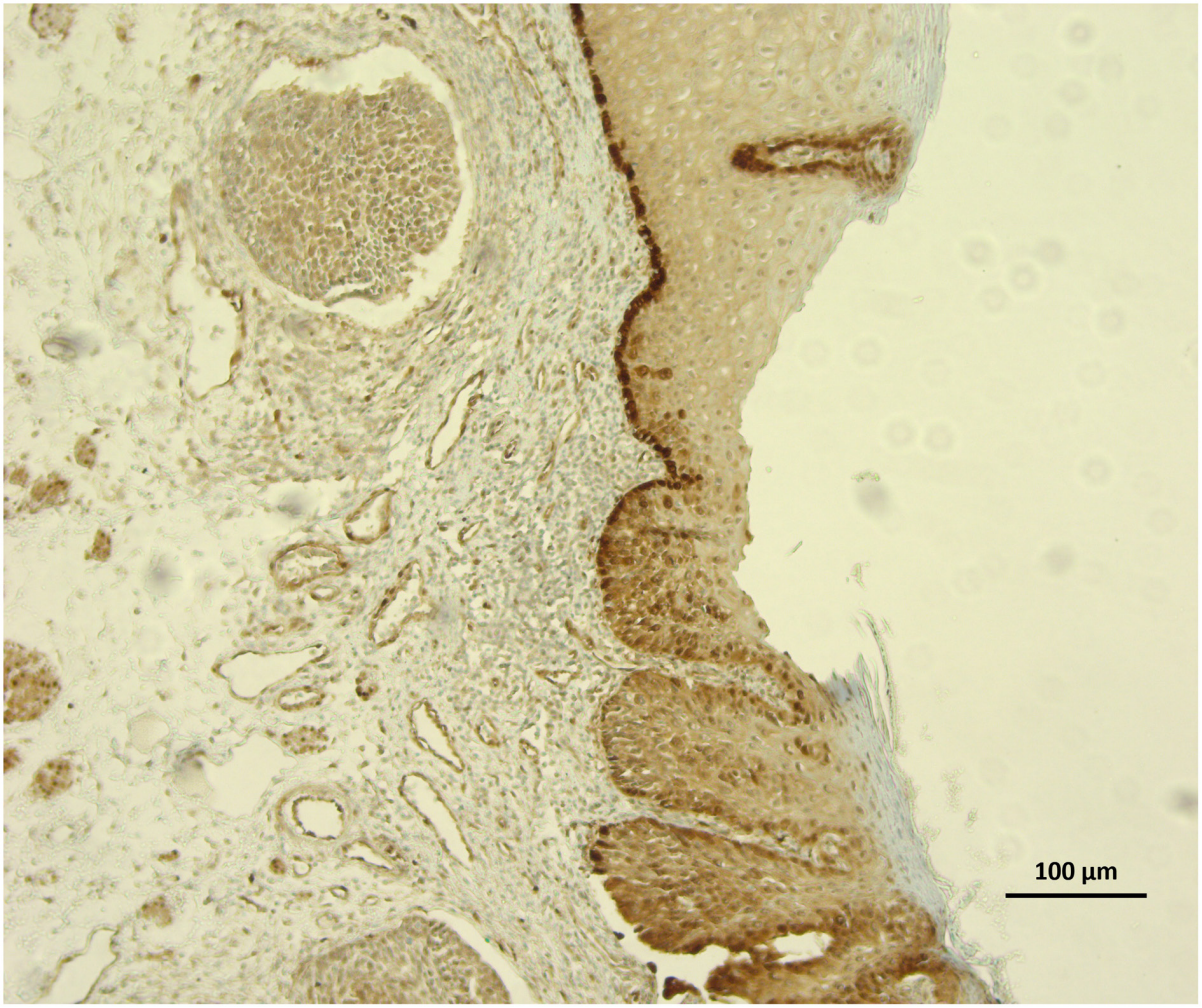
Full coat staining in high-grade epithelial dysplasia areas and only basal layer staining in the adjacent epithelium without dysplasia.

**Figure 5.**
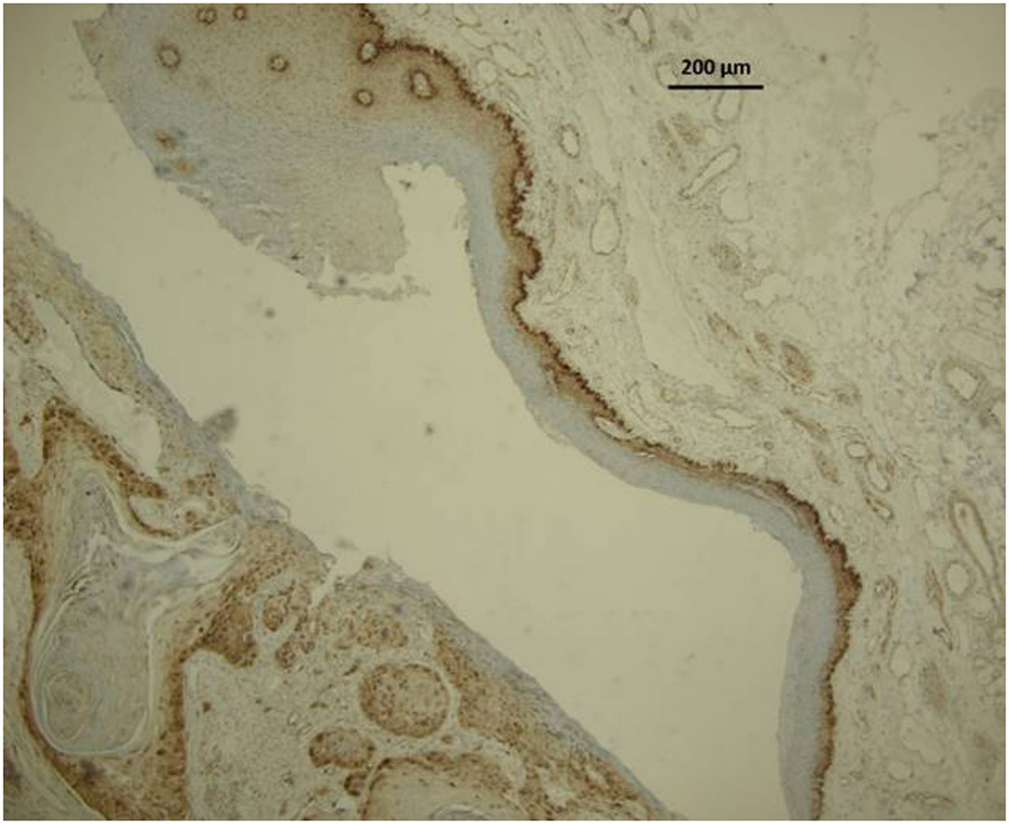
More intense expression in the basal layer of the adjacent epithelium and the periphery of tumor solid islands.

For a total of 104 cases, the paraffin blocks of LSCC and adjacent non-cancerous tissues were analyzed by immunohistochemistry. In all the cases, HSPA-2 positivity was determined in tumoral areas (100%). The overexpression rate of HSPA-2 in adjacent non-cancerous tissues with dysplasia was 48/104 (42%) (*P* < .0001). While positivity was observed only in 1/3 of the basal part of the epithelium in low-grade epithelial dysplasia areas, full coat staining was seen in high-grade epithelial dysplasia areas. The expression of HSPA-2 expression was only observed in the basal layer of adjacent squamous epithelium not containing dysplasia in all the cases. In well-differentiated tumors, a more distinct expression was observed predominately in the periphery of solid tumor islands. In areas where differentiation loss was pronounced, more widespread and severe expression was present (Figures 2-5).

### Prognostic Significance of Heat Shock-Related 70-kDa Protein 2 expression in Laryngeal Squamous Cell Carcinoma

The association between HSPA-2 expression and clinicopathological parameters of patients with LSCC was assessed with the Spearman correlation analysis. The overexpression of HSPA-2 was statistically significantly correlated with prognostic parameters, namely macroscopic tumor size, lymphovascular invasion, pT, pN, pM, TNM stage, and recurrence (*P* < .05). On the other hand, there was no significant association between HSPA-2 expression and histological grade (Table 2). There were also no significant differences in the remaining parameters, that is, peritumoral lymphocytic reaction (*P* = .37), presence of ulceration (*P* = .27), perineural invasion (*P* = .35), age (*P* = .56), and gender (*P* = .35).

**Figure 6.**
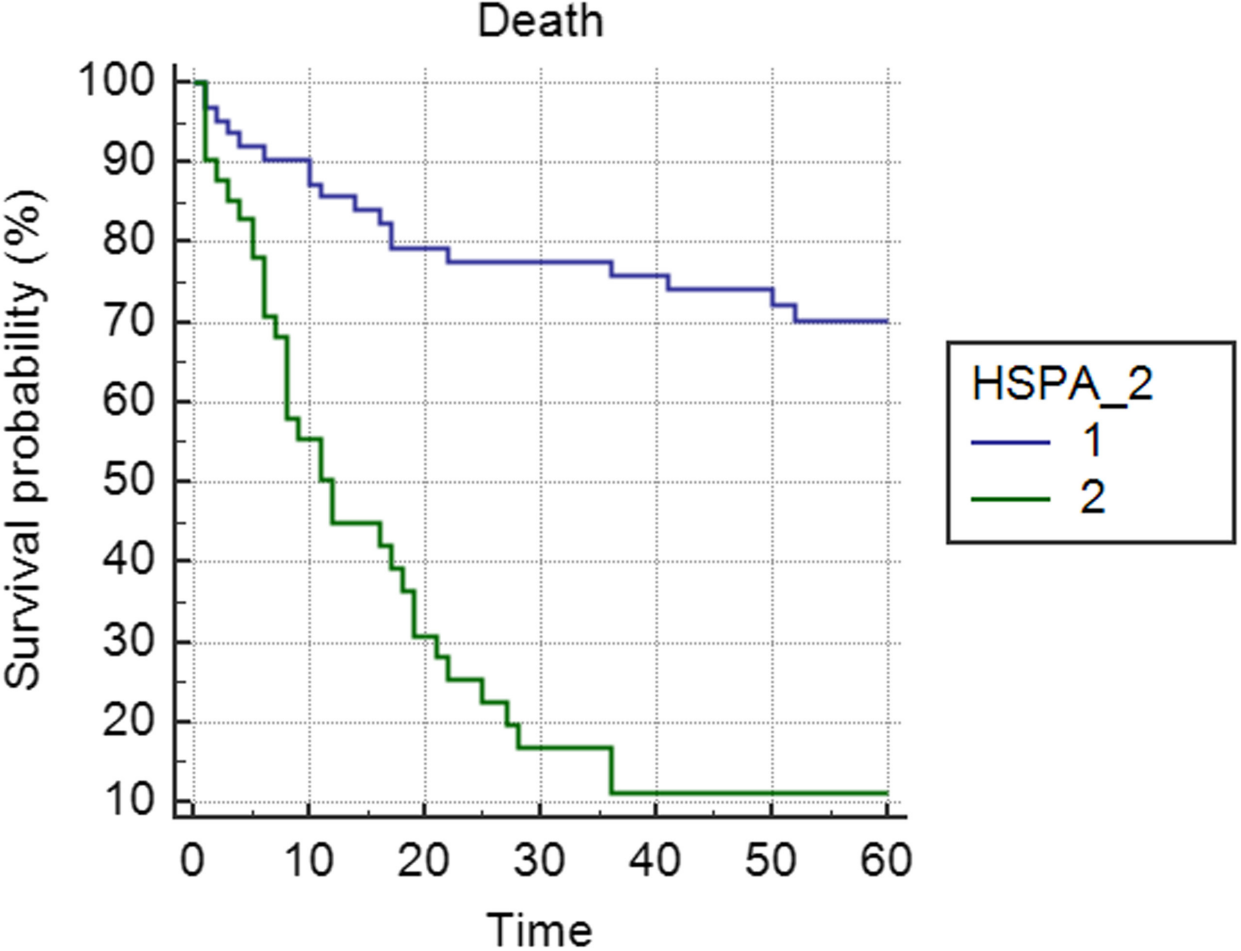
Kaplan–Meier estimate of survival in all patients according to heat shock-related 70-kDa protein 2 expression.

During the survival analysis, HSPA-2 expression was divided into 2 groups as low expression and high expression. Histoscores 1 and 2 were considered as low expression (group 1) and histoscore 3 was considered as high expression (group 2). The Kaplan–Meier survival analysis showed that the 5-year survival rates significantly differed between groups 1 and 2 (71.43% and 19.51%, respectively, *P* < .001) (Figure 6). Overexpression of HSPA-2 was associated with a shorter survival time. The Cox regression multivariate survival analysis including pT, pN, pM, TNM stage, recurrence, and HSPA-2 expression revealed that HSPA-2 expression had a significant correlation with LSCC prognosis, and it was an independent prognostic factor of outcomes in patients with LSCC (hazard ratio = 2.5864, 95% CI = 1.1026-6.0671, *P* = .029) (Table 3).

**Figure 7.**
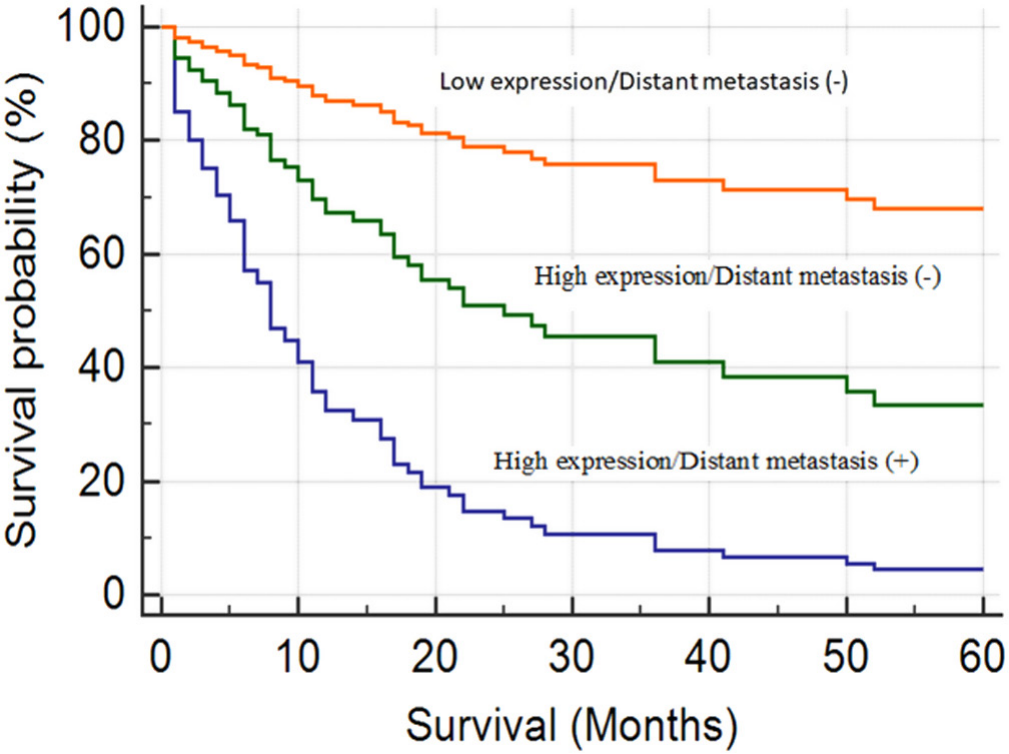
Heat shock-related 70-kDa protein 2 expression/distant metastasis status (Cox regression analysis).

**Figure 8.**
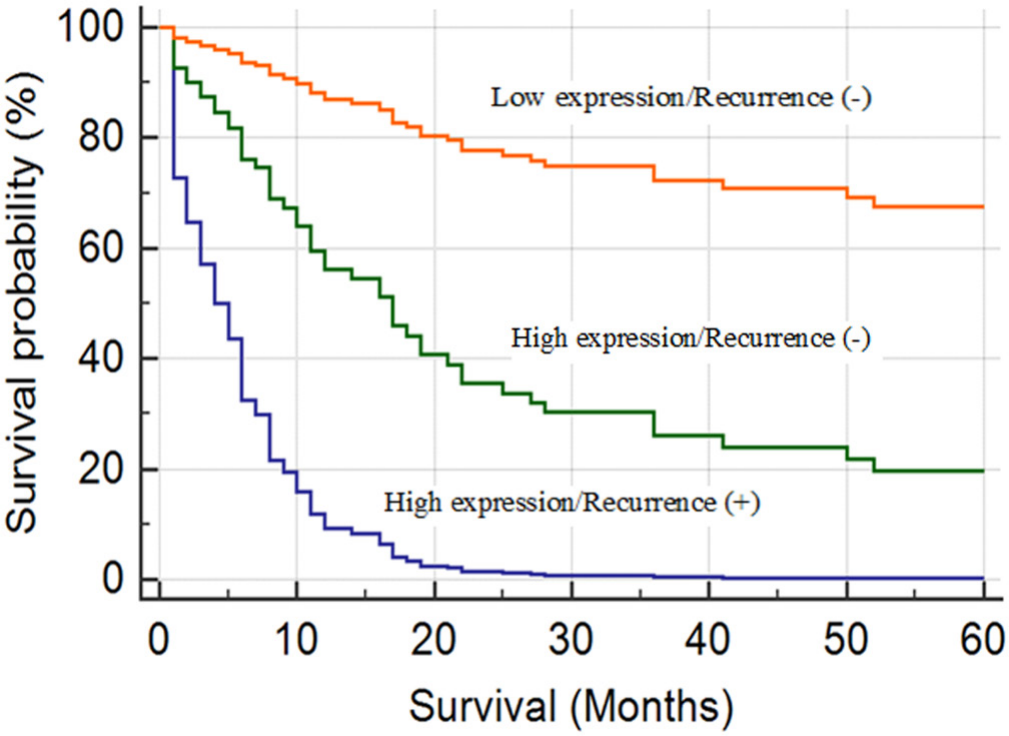
Heat shock-related 70-kDa protein 2 expression/recurrence status (Cox regression analysis).

A subgroup analysis was performed by combining HSPA-2 expression with distant metastasis and recurrence. To obtain survival curves, we divided the patients into 3 groups: high expression/distant metastasis (+)/recurrence (+), high expression/distant metastasis (−)/recurrence (−), and low expression/distant metastasis (−)/recurrence (−). There was no patient with low expression/distant metastasis (+)/recurrence (+). The results showed that the survival time was shorter in the group with high expression/distant metastasis (+)/recurrence (+) than in the remaining 2 groups (*P* < .0001/*P* < .0001). Furthermore, the survival time was shorter in the group with high expression/distant metastasis (−)/recurrence (−) than in the group with low expression/distant metastasis (−)/recurrence (−) (Figures 7 and 8).

## Discussion

Heat shock proteins are chaperon proteins that have protective function for cells by creating an adaptation mechanism and increasing it in the presence of environmental and pathophysiological stressors, and they constitute an important step in the defense system. HSP70t and HSP70-2 (HSPA-2), unlike other HSP70 chaperon proteins, are expressed in testicular tissues, and they are either not seen or are rarely present in other tissues.^19^ In a study conducted on rats, Zhu et al^20^ reported that when they caused HSPA-2 defects, primary spermatocytes were stuck in meiosis I, and other HSPA proteins were not able to compensate for this. Based on these results, the authors argued that HSPA-2 was a molecular chaperone for CDC2/cyclin B1 complex formation and was necessary to ensure G2/M-phase transition during mitosis and meiotic cell cycles. In a different study, it was noted that G1/S phase arrest occurred, and malignant cell proliferation decreased after the HSPA-2 gene was suppressed in lung adenocarcinomas. It was shown that reduced HSPA-2 affected the ERK1/2 cascade that has the functions of cell survival, proliferation stimulation, differentiation, aging, and apoptosis.^21^

It has been reported that HSPA-2 is effective in many malignant carcinogenesis. While it is not expressed in normal tissues, it is excessively expressed in malignant cells.^14^ The excessive expression of HSPA-2 has been shown in malignancies of the pancreas,^7^ cervix,^8^ bladder,^9^ lungs,^14^ esophagus,^15^ nasopharynx,^16^ and liver.^17^ All of these studies show that HSPA-2 is effective in the formation and development of tumors. In our study, we confirmed that HSPA-2 was expressed in LSCC. In all the cases, HSPA-2 positivity was determined in tumoral areas (100%). The overexpression rate of HSPA-2 in adjacent non-cancerous tissues with dysplasia was 48/104 (42%). The expression of HSPA-2 was only observed in the basal layer of adjacent squamous epithelium not containing dysplasia in all the cases. There was a diffused and strong expression in moderately and poorly differentiated tumors while weak staining was seen in the periphery of well-differentiated tumor islands. Similarly, Zhang et al^15^ reported that while they did not observe staining with HSPA-2 in normal esophagus epithelial tissue, they detected poor staining in areas adjacent to the tumor and in the squamous epithelium basal layer and stronger staining in infiltrative areas. In their study, Scieglinska et al^14^ reported observing positive HSPA-2 in keratinocytes in the adjacent epidermis basal layer and infiltrative areas of all skin tumors. On the other hand, they also observed positivity with HSPA-2 in infiltrative areas in half of their cases, while there was no positivity in the normal epithelium in areas adjacent to the tumor in SCC in the head and neck region.

A different finding of our study was the presence of significant differences in staining rate and intensity between low-grade and high-grade epithelial dysplasia areas of the adjacent epithelium. In the low-grade epithelial dysplasia areas of all our cases, positivity was found in only 1/3 of the basal part of the epithelium, while full coat strong staining was seen in high-grade epithelial dysplasia areas. Based on this different staining pattern, we consider that the HSPA-2 expression pattern can be extremely helpful in the differential diagnosis when distinguishing epithelial dysplasia in laryngeal biopsies. No study has been conducted in the literature on this subject.

In addition to HSPA-2 being excessively expressed in different types of cancer, there are a few studies showing that it has an effect on tumor progression.^11-17^ It is indicated that tumor cells gain resistance against apoptosis related to hypoxia with an increase of HSPA-2, and that is how they stay alive.^22^ It is also known that HSPA-2 migrates to the nucleus and nucleoli under stress conditions while it is localized in the cytoplasm under normal conditions. This is critical for cell migration, tumor formation, and metastasis since HSPA-2 moves continuously between the cytoplasm and the nucleus in a heat shock state.^23^ In our study, HSPA-2 staining in the cytoplasm and nucleus was observed in the infiltrative areas.

A significant relationship was observed between the excessive expression of HSPA-2 and important prognostic parameters such as macroscopic tumor size, lymphovascular invasion, pT, pN, pM, TNM stage, recurrence, and survival rates. There was also an association between survival and HSPA-2 expression. With the Kaplan–Meier analysis of overall survival, we found that patients with increased HSPA-2 expression at all stages had shorter survival regardless of other prognostic parameters. In addition, the Cox regression multivariate survival analysis proved that HSPA-2 expression had a significant correlation with the LSCC prognosis, and it was an independent prognostic factor of outcomes in patients with LSCC. In addition, with our results of the Cox regression, multivariate survival analysis indicated that distant metastasis and recurrence had a significant correlation with survival, and they were independent prognostic factors of LSCC. Therefore, a subgroup analysis was performed by combining HSPA-2 expression with distant metastasis and recurrence, and the results showed that the group with high expression/distant metastasis and recurrence had a shorter survival time than the other 2 groups. In addition, the survival time was compared according to the HSPA-2 expression level in patients with no distant metastasis and recurrence, and it was found to be shorter in those with high expression. Similar to our study, Zhang et al^22^ stated that HSPA-2 overexpression was associated with pT, clinical stage, pN, and recurrence in esophageal SCC. Similar to our study, Garg et al.^8,9^ Scieglinska et al.^14^ and Fu et al^17^ found a significant correlation between HSPA-2 expression and TNM stage + survival in cervix and bladder carcinomas, non-small cell lung carcinomas, and hepatocellular carcinomas, respectively. Furthermore, Scieglinska et al^14^ highlighted that especially in stage I and II cases, high HSPA-2 expression was related to lower survival rates. Unlike our study, all the authors mentioned above observed a significant relationship between histological grade and HSPA-2 expression. We attribute this difference to the unequal histological grade distribution in our study and the histological grade being moderately differentiated (G2) in most of our cases. In another similar study, Fu et al^17^ reported that in hepatocellular carcinomas, the tumor radius was directly proportionate to HSPA-2. Zhai et al^7^ also noted that HSPA-2 was related to a poor prognosis in tumor angiogenesis and pancreatic adenocarcinoma and played an important role in its progression. Various studies have shown that cases presenting with an excessive expression of HSPA-2 had higher pN rates and thus a worse prognosis.^12,23^ In their in vivo study using a mouse model, Garg et al^8^ considered the matter from a different point of view and stated that tumor growth was reduced by 75% with HSPA-2 inhibition in rats and they argued that HSPA-2 expression was related to cellular motility and growth, playing a role in cancer spread during early stages.

Evaluating the results of our study and the available data in the literature together, we consider that HSPA-2 can be involved in carcinogenesis, tumor development, progression, and metastasis. The presence of a significant relationship between HSPA-2 expression and important prognostic parameters such as macroscopic tumor size, lymphovascular invasion, pT, pN, pM, TNM stage, and survival rate suggests that HSPA-2 can be used as prognostic marker in LSCC with an immunohistochemical analysis. Our study showed, for the first time, that HSPA-2 could be an important prognostic indicator in LSCC.

Some limitations should be considered in our study. Although our sample size and follow-up period seem to be sufficient, our results should be supported by studies conducted with larger populations and longer observation periods.

In conclusion, our study supports the presence of an association between high HSPA-2 expression levels and prognostic parameters in LSCC. We consider that HSPA-2 can be used as a prognostic marker in LSCC. In addition, it may be important in early diagnosis due to the increased expression of HSPA-2 even under LSCC precursor conditions. Although our findings are remarkable, since there are no other studies investigating the relationship between LSCC and HSPA-2 expression and prognosis, there is a need to support our findings in future work.

## Figures and Tables

**Table 1.** Demographic and Histopathological Features of the Patients

	Patients (n = 104)
**Age**	56 ± 8.1
**Gender n (%)**	
*Male*	94 (90.3)
*Female*	10 (9.7)
**Tumor macroscopic diameter (cm), n (%)**	3.1 ± 1.8 (0.8-6 cm)
≥3 cm	64 (61.5)
< 3 cm	40 (42.5)
**Lymphovascular invasion, n (%)**	
*Not identified*	63 (60.5)
*Present*	41 (39.5)
**Peritumoral lymphocytic reaction, n (%)**	
*Mild*	27 (25.9)
*Moderate*	50 (48.2)
*Severe*	27 (25.9)
**Histologic grade, n (%)**	
*Well differentiated (G1)*	28 (26.9)
*Moderately differentiated (G2)*	63 (60.6)
*Poorly differentiated (G2)*	13 (12.5)
**pT, n (%)**	
*pT1*	12 (11,5)
*pT2*	18 (17.3)
*pT3*	31 (29.9)
*pT4*	43 (41.3)
**pN, n (%)**	
*pN0*	70 (67.3)
*pN1*	26 (25)
*pN2*	6 (5.8)
*pN3*	2 (1.9)
**pM, n (%)**	
*pM0*	81 (77.9)
*pM1*	23 (22.1)
**TNM stage n (%)**	
*Stage I*	12 (11.5)
*Stage II*	16 (15.4)
*Stage III*	21 (20.2)
*Stage IV*	55 (52.9)
**Recurrence, n (%)**	
*Not identified*	96 (93)
*Present*	8 (7)
**Outcome n (%)**	
*Survived*	53 (50.9)
*Died*	51 (49.1)

pT, primary tumor; pN, lymph node metastasis; pM, distant organ metastasis.

**Table 2.** Relationship Between Prognostic Factors and HSPA-2 Expression (Spearman’s Correlation Test)

	**Histoscore**				*P* *r*
	Grade 0 (n = 0)	Grade 1 (n = 22)	Grade 2 (n = 41)	Grade 3 (n = 41)	
**MTV (n)**					
V ≥3 cm	0	8	27	29	*.0092*
V < 3 cm	0	14	14	12	*0.254*
**LVI (n)**					
*Not identified*	0	18	15	8	*<.0001*
*Present*	0	4	26	33	*0.442*
**pT (n)**					
*pT1*	0	12	0	0	
*pT2*	0	8	6	4	*<.0001*
*pT3*	0	2	18	11	*0.606*
*pT4*	0	0	17	26	
**pN (n)**					
*pN0*	0	22	27	21	
*pN1*	0	0	10	16	*.0002*
*pN2*	0	0	4	2	*0.355*
*pN3*	0	0	0	2	
**pM (n)** *Not identified*	0 0	22 0	41 0	18 23	*<.0001* *0.600*
*Present*					
**TNM stage n (%)**					
*Stage I*	0	12	0	0	
*Stage II*	0	8	6	2	*<.0001*
*Stage III*	0	2	14	5	*0.688*
*Stage IV*	0	0	21	34	
**Recurrence n (%)**					
*Not identified*	0	22	41	34	*.0018*
*Present*	0	0	0	7	*0.303*
**HG (n)**					
*G1*	0	6	10	12	*0.4186*
*G2*	0	16	26	21	*0.0802*
*G3*	0	0	5	8	

HSPA-2, heat shock-related 70-kDa protein 2, MTV, macroscopic tumor volume; LVI, lymphovascular invasion; pT, primary tumor; pN, lymph node metastasis; pM, distant organ metastasis; HG, histological grade; G1, well; G2, moderately; G3, poorly differentiated.P value <.05 was accepted as significant.

**Table 3.** Univariate and Multivariate Cox Regression Analyses of the Most Important Prognostic Factors in Patients with LSCC

	**Univariate Analysis**		**Multivariate Analysis**	
**95% CI**	*P*	**95% CI**	*P*
**Primary tumor** (T3 + T4/T1 + T2)	4.4962-76.2354	.0001	0.3196-7.3493	.5953
**Lymph node metastasis** (+/−)	2.3093-7.1824	<.0001	1.0831-4.2539	**.0294**
**Distant metastasis** (+/−)	3.0423-9.9135	<.0001	0.4463-2.4384	.9226
**TNM stage** (III+IV/I + II)	5.23863E-166-31.99967E+177	.9402	2.71575E-168-759.62894E+177	.9449
**Recurrence**	2.8225-15.2173	<.0001	1.4573-13.3682	**.0090**
**HSPA-2 expression** (high/low)	3.1524-10.6815	<.0001	1.1026-6.0671	**.0298**

LSCC, laryngeal squamous cell carcinoma; HSPA-2, heat shock-related 70-kDa Protein 2.P value <.05 was accepted as significant.
